# GOLM1 promotes prostate cancer progression via interaction with PSMD1 and enhancing AR‐driven transcriptional activation

**DOI:** 10.1111/jcmm.70186

**Published:** 2024-10-29

**Authors:** Guang Yan, Tianhang Zhu, Jiawei Zhou, Xia Li, Zonghua Wen, Bahaerguli Miuhuitijiang, Zhiyong Zhang, Yuejun Du, Chengyao Li, Xiaojun Shi, Wanlong Tan

**Affiliations:** ^1^ Department of Urology, Nanfang Hospital Southern Medical University Guangzhou China; ^2^ Department of Andrology, Shanghai Seventh People's Hospital Shanghai University of Traditional Chinese Medicine Shanghai China; ^3^ State Key Laboratory of Holistic Integrative Management of Gastrointestinal Cancers, Department of Biochemistry and Molecular Biology The Fourth Military Medical University Xi'an Shaanxi China; ^4^ Department of Pathology Shenzhen University General Hospital Shenzhen China; ^5^ Department of Transfusion Medicine, School of Laboratory Medicine and Biotechnology Southern Medical University Guangzhou China

**Keywords:** AR, GOLM1, prostate cancer, PSMD1, UPS

## Abstract

Aberrant transcriptional activation of the androgen receptor (AR) is a predominant cause of prostate cancer (PCa), including both in the initial and androgen‐independent stages. Our study highlights Golgi membrane protein 1 (GOLM1) as a key regulator of AR‐driven transcriptional activity in PCa progression. Utilizing local clinical data and TCGA data, we have established a robust association between GOLM1 and AR target genes, and further demonstrated that GOLM1 can enhance the expression of AR target genes. We discovered that GOLM1 interacts with PSMD1, a component of the 19S regulatory complex in the 26S proteasome, using mass spectrometry and Co‐IP analysis. It is well known that ubiquitin‐proteasome plays a vital role in AR expression and transcriptional regulation. Our findings demonstrate that GOLM1 enhances ubiquitin proteasome activity by binding to PSMD1, thereby facilitating AR‐driven transcriptional activity and PCa progression. These results indicate that GOLM1 and its associated proteins may become potential therapeutic targets for PCa characterized by dysregulated AR‐driven transcriptional activation.

## 1 INTRODUCTION

Prostate cancer (PCa) is the second most common cancer in males to be diagnosed, and it is the fifth largest cause of cancer‐related mortality globally.^1^ As a typical nuclear receptor, the main function of androgen receptor (AR) is to bind to its corresponding ligand, and further activate downstream target genes expression. This function is essential to the development and course of PCa.^2^ When a PCa patient suffers from locally advanced, metastatic or biochemical recurrence, androgen deprivation therapy (ADT) is the primary choice.^3^ Even though ADT has been shown to have some early benefits, it is linked to the expected establishment of resistance, which can result in the development of castration‐resistant prostate cancer (CRPC).^4^ AR‐driven transcriptional activity has been observed to undergo significant alterations in CRPC, leading to the upregulation of numerous AR‐regulated genes as the disease progresses to CRPC.^4^, ^5^ These findings provide evidence for the continued activation of AR signalling during this stage of the disease. Thus, it is important to investigate the molecular mechanisms behind the start of AR signalling and how they affect the emergence of androgen depletion resistance.

Recently, newer agents like enzalutamide (an AR signalling inhibitor)^6^ and abiraterone acetate (a CYP17A1 inhibitor)^7^ have been introduced that target‐specific mechanisms of resistance, offering additional survival advantages. However, it is important to note that these agents will eventually prove ineffective in suppressing CRPC.^8^ Although some of the mechanisms underpinning these agents' failure are distinct, many are similar to those that promote the progression of CRPC. A thorough comprehension of the processes resulting in aberrant transcriptional activation of AR will greatly assist in guiding future research and therapeutic strategies.

Golgi membrane protein 1 (GOLM1) was first shown to be a biomarker for PCa in 2008.^9^ Subsequent investigations have revealed its elevated expression and pro‐cancer properties in other cancer types, including hepatocellular carcinoma,^10^ oesophageal carcinoma,^11^ lung cancer,^12^ and glioma.^13^ Research on hepatocellular carcinoma has indicated that GOLM1 regulates EGFR/RTK recycling to encourage liver cancer cells to proliferate and spread.^10^ Additionally, our previous findings demonstrate that GOLM1 plays a pro‐cancer role in PCa by activating the PI3K‐Akt signal.^14^ Interestingly, our latest research has found that GOLM1 has a pro‐cancerous function in PCa due to its involvement in transcriptional regulation of AR signalling. We further confirmed that GOLM1 increases proteasome activity by interacting with proteasome 26S subunit, non‐ATPases 1 (PSMD1), thereby promoting AR‐driven transcriptional activation. For people with PCa, particularly those who have not responded to ADT, our research may offer new treatment targets.

## 2 MATERIALS AND METHODS

### 2.1 PCa patients' samples

Tissue microarrays (TMAs) containing 34 PCa patients' samples (89 points: 27 Paracancer, 22 Gleason score 3, 22 Gleason score 4 and 18 Gleason score 5) were acquired from the Department of Pathology of Nanfang Hospital; 31 RNA samples and six IHC samples were obtained from PCa patients during surgery in the Department of Urology of Nanfang Hospital. Nanfang Hospital Research Ethics Committee at Southern Medical University approved the research, and all patients gave their informed agreement.

### 2.2 Cell lines

Human AR prositive PCa cell lines C4‐2 [RRID: CVCL_4782], LNCaP [RRID:CVCL_0395], and 22Rv1 [RRID: CVCL_1045] were purchased from Chinese Academy of Sciences Cell Bank (Shanghai, China), and cultured in RPMI‐1640 purchased from Invitrogen (NY, USA) supplemented with 10% FBS (Gibco, Australia). Cells were grown at 37°C in incubators with humidified atmospheres of 5% CO_2_ and 95% air.

### 2.3 Reagents and antibodies

Enzalutamide, Dihydrotestosterone and Cycloheximide were purchased from MedChemExpress (Princeton, NJ, USA). Anti‐GOLM1 (Rabbit) was purchased from Abcam (Cambridge, MA, USA). Anti‐GOLM1 (Mouse) and anti‐PSMD1 were procured from Sata Cruz Biotechnology located in Dallas, Texas, USA. Antibodies against AR, KLK3, Ub and β‐actin were procured from Cell Signaling Technology, located in Beverly, Massachusetts, in the United States.

### 2.4 Vectors and lentivirus

The *GOLM1* (ID: NM_016548.3) CDS (1203 bp) was amplified by PCR and cloned into pLEX‐Flag‐MCS vector (Thermo Scientific, Waltham, MA, USA) with restriction enzymes BamHI and NotI (New England Biolabs, Ipswich, MA, USA). Lentivirus packaging was performed as previously described.^14^ XhoI and KpnI restriction enzymes (New England Biolabs, Ipswich, MA, USA) were used to insert the *KLK3* (ID: ENSG00000142515) promoter (−1 to −641) into the pGL3‐Promoter vector (Promega, Madison, WI, USA) after it had been amplified by PCR. The KLK3 promoter sequences were listed in Table S1. Following lentivirus infection and puromycin (1 μg/mL) selection, the stable cell lines LNCaP‐GOLM1 and C4‐2‐GOLM1 were obtained. We performed transient transfections in cells using lipofectamine 3000 (Promega, Madison, WI, USA) in accordance with the instructions of manufacturer.

### 2.5 Pan‐cancer analysis, overall survival analysis and scatter‐correlation analysis

Pan‐cancer RNA sequencing data were acquired via the UCSC Xena data portal.^15^ Data on the FPKM RNA‐Seq of 495 prostate adenocarcinoma (PRDA) patients' tissues and match 52 normal tissues were downloaded from the TCGA database. Pan‐cancer analysis, overall survival analysis and scatter‐correlation analysis were performed by the ‘UCSCXenaShiny’ R package.^16^

### 2.6 Cell proliferation, invasion and migration assays

The Cell Counting Kit‐8 (Dojindo Laboratories, Japan) and the 5‐ethynyl‐2‐deoxyuridine (EdU) kit (Beyotime, Jiangsu, China) were used to assess cell proliferation following to the instructions. Cell invasion was assessed by Transwell assay which needs prepare 8.0 μm Transwell insert (MiliporeMilicell, Billerica, MA) and Matrigel (BD Bioscience, Oxford, UK). Using the wound‐healing assay, cell migration was measured. Detailed experimental protocols performed as previously described.^14^

### 2.7 In vivo xenograft assay

Male nude mice were bred and raised at the Animal Center of Southern Medical University (six in each group, a total of 12 mice). At 6 weeks of age, male nude mice were inoculated subcutaneously in the limb with either 1 × 107 C4‐2‐Vector or C4‐2‐GOLM1 cells. The growth of the tumour was evaluated by monitoring the dimensions of the tumour every 10 days over a period of 50 days with vernier callipers, and the volume of the tumour was determined by applying the formula: volume (mm^3^) = length (mm) × width (mm^2^) × 0.5. Following sacrifice, the tumour weight of the mice was recorded. Additionally, the proliferation of tumour cells was evaluated through Ki67 staining (Cell Signaling Technology, Beverly, MA, USA). Nanfang Hospital Animal Ethics Committee of Southern Medical University approved the animal experiment protocols.

### 2.8 RNA interference and real‐time qRT‐PCR analysis

Every siRNA duplex was purchased from GenePharma, located in Suzhou, China. The specific siRNA sequence of GOLM1 or PSMD1 were described in Table S2. Transfection of siRNA were carried out by lipofectamine 3000 according to manufacturer's instructions. qRT‐PCR analysis was conducted following to the previous description.^17^ A list of the qRT‐PCR primers was found in Table S3.

### 2.9 Luciferase reporter assays

The stable cells LNCaP‐Vector, LNCaP‐GOLM1, C4‐2‐Vector and C4‐2‐GOLM1 grown on 96‐well plates were transiently transfected using lipofectamine 3000 (Promega, USA) with the pGL3‐KLK3 Promoter vector. 48 h following transfection, luciferase activity assays were carried out using a Dual Luciferase Assay Kit purchased from Promega, located in Madison, USA.

### 2.10 Western blot analysis

Western blot analysis was fulfilled in accordance with our previous report.^17^ Briefly, probed with anti‐GOLM1 (Abcam 1:1500), anti‐GOLM1 (Sata Cruz 1:500), anti‐AR (CST 1:2000), anti‐KLK3 (CST 1:1000), anti‐Ub (CST 1:1000), anti‐PSMD1 (Sata Cruz 1:500) and Anti‐β‐Actin (CST 1:2000), followed by secondary antibodies (1:5000). Using an ECL (Bio‐Rad, USA) system, protein bands were detected.

### 2.11 Immunohistochemistry

A standard immunohistochemistry procedure was followed. Primary antibodies anti‐GOLM1 (Abcam 1:500), anti‐AR (CST 1:600), anti‐KLK3 (CST 1:200) and anti‐PSMD1 (Sata Cruz 1:200) were used, staining with VECTASTAIN® ABC‐HRP Kits (Vector Laboratories, San Francisco, CA, USA). Analysis of protein expression by immunoreactivity score. Total score = Cell score × Colour score. The cell score criterion used the number of cell positive staining (≤5%: 0, 6%–25%: 1, 25%–50%: 2, 51%–75%: 3, ≥75%: 4). Colour score criteria used staining intensity (colourless: 0, mild: 1, moderate: 2 and Strong: 3).

### 2.12 Co‐immunoprecipitation and mass spectrometry analysis

We incubated 2 μg anti‐GOLM1 or 2 μg mouse IgG with 1 mg protein from LNCaP‐GOLM1 or C4‐2‐GOLM1 cell lysates for 6 h at 4°C. Then, overnight, at 4°C, incubate with protein A/G PLUS‐Agarose IP beads (Santa Cruz, CA, USA). After washing with washing buffer, spending 10 min on boiling IP beads in SDS sample buffer. Samples tested by immunoblot with anti‐GOLM1 and anti‐PSMD1.IP samples for mass spectrometry analysis after silver staining, and procedure of mass spectrometry analysis was carried out by Shanghai Applied Protein Technology Co., Ltd.

### 2.13 Immunofluorescence staining and co‐localization assay

After 48 h of culturing on confocal dishes, the cells underwent fixation, permeabilization and stainning with primary antibodies anti‐GOLM1 (Abcam 1:500), anti‐GOLM1 (Sata Cruz 1:200), anti‐AR (CST 1:600) or anti‐PSMD1 (Sata Cruz 1:200) followed by secondary antibody conjugated with Cy3 or Alexa 488 (GeneTex, San Antonio, Texas). Images were captured using confocal microscopy (Olympus Fluoview FV1000) after counterstaining with DAPI (1:5000) for 10 min.

### 2.14 Statistical analysis

Pan‐cancer analysis, overall survival analysis and scatter‐correlation analysis were conducted with RStudio and its appropriate packages. Data were showed as mean ± SEM for independent groups. An ANOVA or a Student's *t*‐test was employed to assess the significance of the findings, and *p* < 0.05 values were regarded as statistically significant. All results were confirmed in at least three independent experiments.

## 3 RESULTS

### 3.1 GOLM1 expression correlates with PCa progression

**FIGURE 1:**
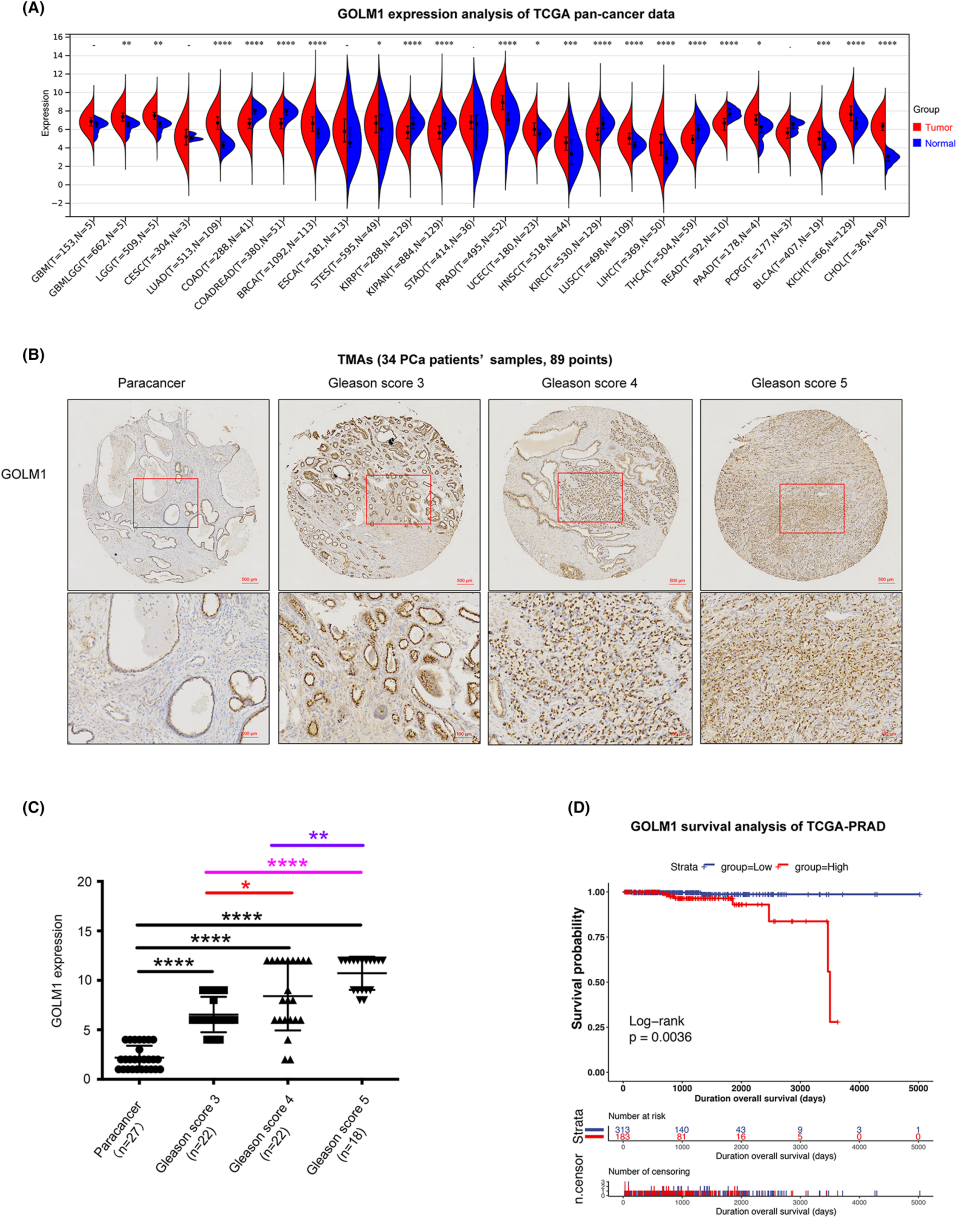
GOLM1 expression is correlated with the PCa progression and its poor prognosis. (A) Pan‐cancer analysis of GOLM1 expression using TCGA data. (Pan‐cancer GOLM1 expression data were acquired via the UCSC Xena data portal, performed log2 (x + 0.001) transformation on each expression value, and calculated by non paired Wilcoxon Rank Sum and Signed Rank Tests. *p < 0.05, **p < 0.01, ***p < 0.005, ****p < 0.001). (B) Immunohistochemical staining of TMA was used to assess GOLM1 expression in paracancer and PCa tissues of Glenn score 3, 4 and 5. Scale bar, 100 μm or 500 μm. (C) Relative expression level of GOLM1 in paracancer tissues and PCa tissues of gleason score 3, 4 and 5 grades (Data were means ± SEM. *p* values were calculated by one‐way ANOVA with Tukey's tests, *p < 0.05, **p < 0.01 and ****p < 0.001). (D) Kaplan–Meier survival curve on GOLM1 expression status in PCa patients. TCGA‐PRAD (496 canses), Kaplan–Meier analysis and log‐rank test were performed by the ‘UCSCXenaShiny’ R package.

A pan‐cancer analysis was conducted on the expression of GOLM1 using TCGA data. It can be found according to the results GOLM1 is upregulated in various tumours, including PCa, lung cancer, liver cancer, colon cancer and breast cancer. Furthermore, its expression is significantly higher in PCa compared to other tumours (Figure 1A). Subsequently, to further clarify the relationship between GOLM1 and the progression of PCa, cancerous and adjacent tissues were collected from 34 PCa patients with varying Gleason scores, and tissue microarrays (TMAs) were constructed. Immunohistochemistry staining of the TMAs revealed a noteworthy elevation in GOLM1 expression in tandem with the escalation of PCa Gleason score (Figure 1B,C). TCGA‐PRAD data was analysed to examine the relationship between overall survival and GOLM1 expression. The findings demonstrated that prostate cancer patients with elevated levels of GOLM1 exhibited a decreased overall survival rate (Figure 1D). These results suggest a direct association between increased GOLM1 expression and the advancement of PCa, leading to a less favourable prognosis.

### 3.2 GOLM1 promotes the malignant phenotype of PCa cells

**FIGURE 2:**
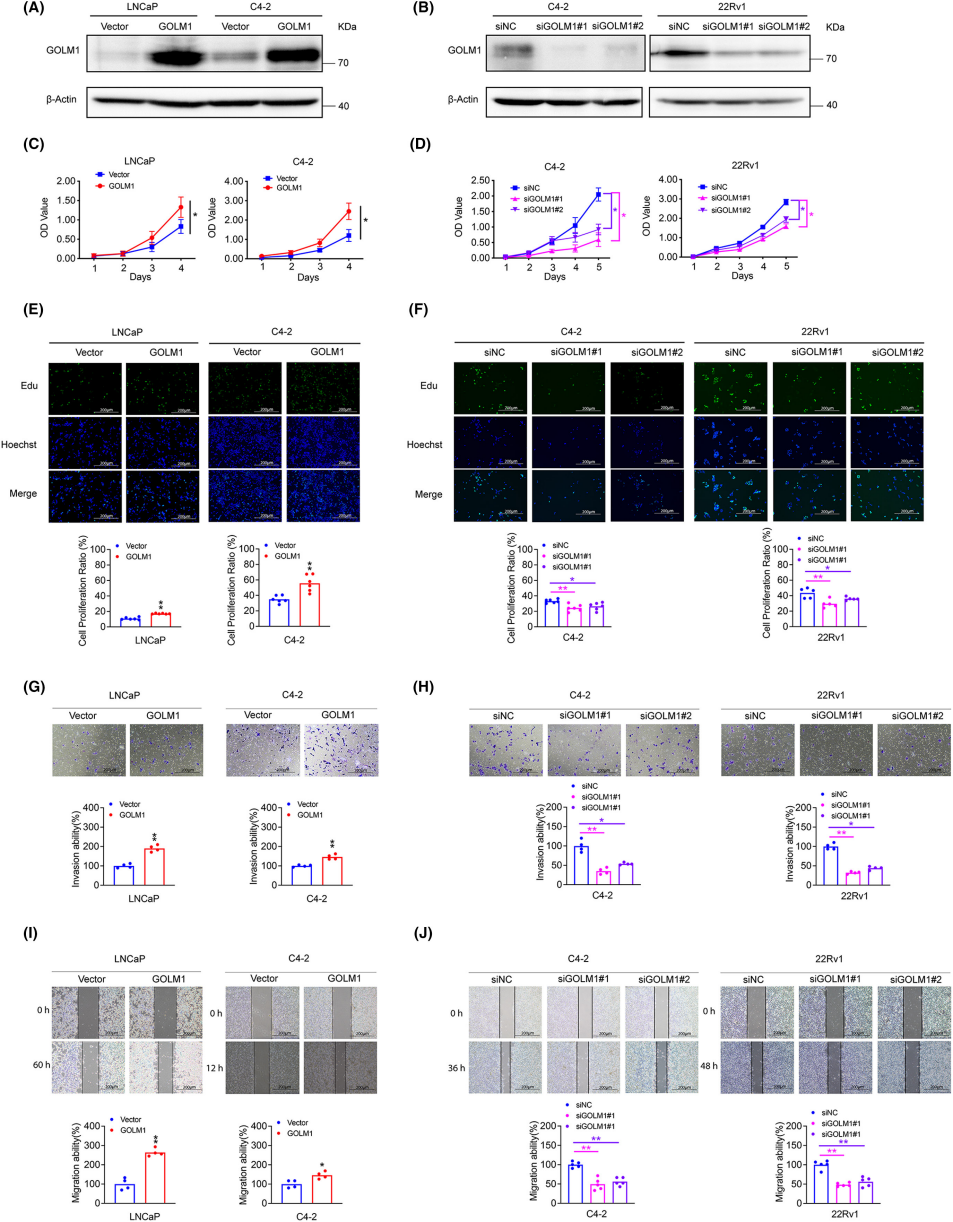
GOLM1 promotes PCa cell proliferation, invasion and migration. (A, B) Western blot analysis of GOLM1 protein expression in GOLM1‐overexpressing cells, GOLM1‐silencing cells and control cells. (C– F) CCK8 assays and EdU assays were used to determine the proliferation ability of GOLM1‐overexpressing cells, GOLM1‐silencing cells and control cells. (G–J) Transwell assays analysis of cell invasion ability and Wound healing assays analysis of cell migration ability were performed in the GOLM1‐overexpressing cells, GOLM1‐silencing cells and control cells. Data were means ± SEM. *p* values were calculated by two‐way ANOVA with Sidak multiple comparisons tests (C, D); *p* values were calculated by two‐tailed t‐test (E, G, I); *p* values were calculated by one‐way ANOVA with Dunnett's multiple comparisons tests. (F, H, J) *p < 0.05, **p < 0.01. Scale bar, 200 μm.

Based on the aforementioned data, we conducted an evaluation of GOLM1's functions in PCa cells to determine its potential significance in the progression of PCa. Initially, we generated stable PCa cell lines overexpressing GOLM1 (LNCaP‐GOLM1 and C4‐2‐GOLM1) through lentivirus infection, and subsequently depleted GOLM1 using small interfering RNAs specific to GOLM1 (siGOLM1#1 and siGOLM1#2) via transfection. The levels of GOLM1 expression were validated through Western blot and quantitative RT‐PCR analyses (Figure 2A,B and Figure S1A,B). CCK8 and EdU assay results indicate that overexpression of GOLM1 prominently enhances the proliferation capacity of PCa cells LNCaP and C4‐2 (Figure 2C,E), while silencing GOLM1 reduces the proliferation ability of PCa cells C4‐2 and 22Rv1 (Figure 2D,F). Furthermore, transwell and wound‐healing assays reveal that GOLM1 upregulation promotes invasion and migration in PCa cells LNCaP and C4‐2 (Figure 2G,I), whereas GOLM1 silencing weakens invasion and migration in PCa cells C4‐2 and 22Rv1 (Figure 2H,J). These gain‐of‐function and loss‐of‐function studies conducted in vitro provide evidence that GOLM1 is implicated in promoting the malignant phenotype of PCa cells.

### 3.3 GOLM1 promotes the growth of PCa in xenograft mice models

**FIGURE 3:**
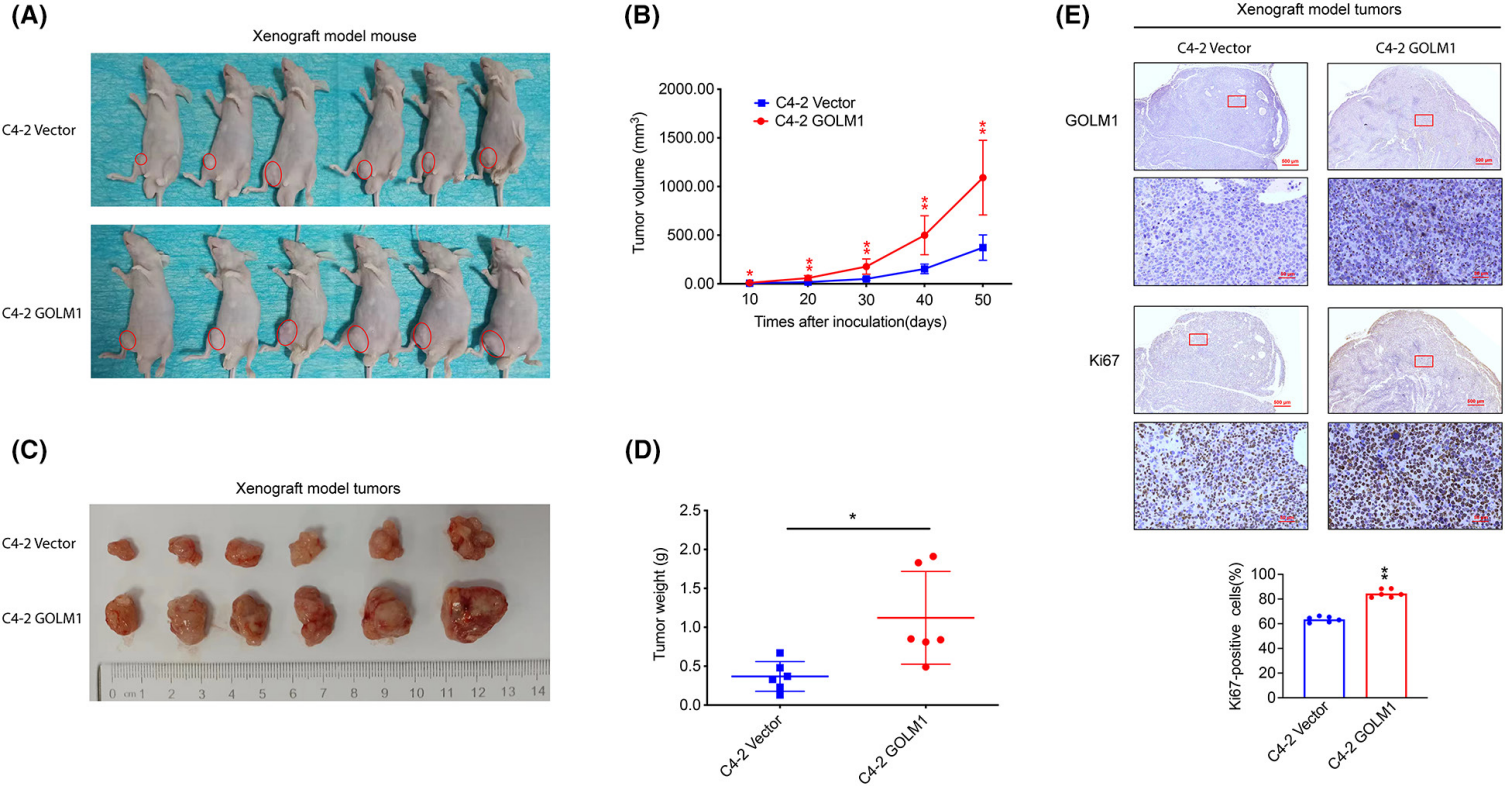
GOLM1 promotes PCa tumour growth in vivo. (A, B) Subcutaneous tumours and growth curve of xenograft models derived from C4‐2‐Vector and C4‐2‐GOLM1. (C, D) Appearance and weight of tumours after 50 days. (E) GOLM1 and Ki‐67 expression of xenograft tumours were reflected by immunostaining. Data were means ± SEM. *p* values were calculated by two‐way ANOVA with Sidak multiple comparisons tests (B); *p* values were calculated by Mann–Whitney test (D); *p* values were calculated by two‐tailed *t*‐test (E). *p < 0.05, **p < 0.01. Scale bar, 50 μm or 500 μm.

After inoculating PCa cells into xenograft mice models, GOLM1's tumour‐promoting function was further validated. The results depicted in Figure 3A,B demonstrate that nude mice injected with C4‐2‐GOLM1 cells exhibited accelerated tumour growth compared to those injected with C4‐2‐Vector cells. Finally, compared to mice injected with C4‐2‐Vector cells, nude mice injected with C4‐2‐GOLM1 cells produced noticeably larger tumours (Figure 3C,D). In addition, Ki67 staining results revealed that GOLM1 overexpression enhances PCa cell proliferation (Figure 2E). Overall, these data further verified GOLM1's oncogenic function in PCa.

### 3.4 GOLM1 is involved in regulation of AR‐driven transcriptional activity and its nuclear expression

**FIGURE 4:**
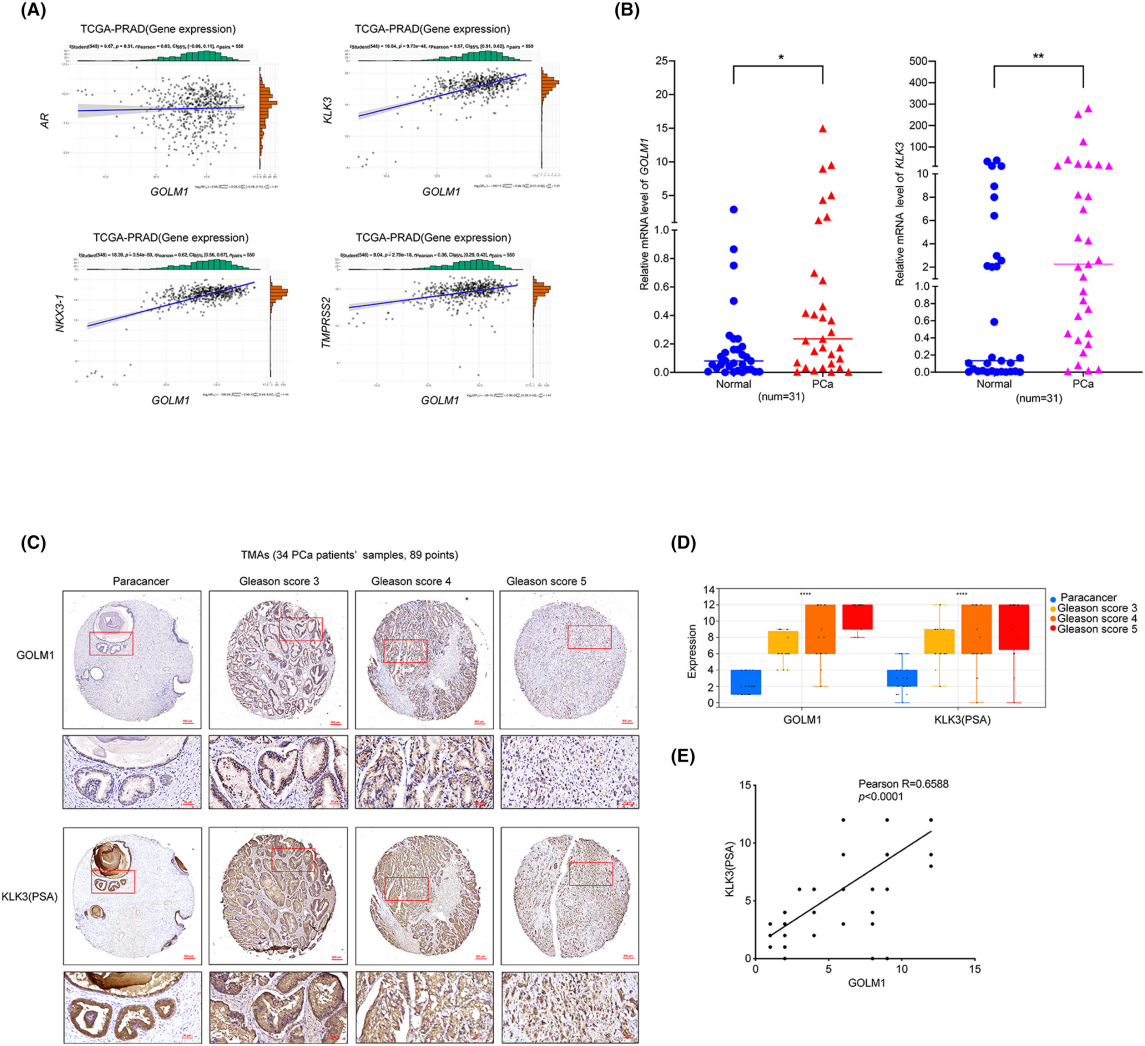
GOLM1 has a close correlation with AR signalling. (A) Scatter‐correlation analysis between GOLM1and AR as well as its target genes (*KLK3*, *NKX3‐1* and *TMPRSS2*) based on the TCGA‐PRAD data. (B) Real‐time RT‐PCR analysis of *GOLM1* and *KLK3* mRNA level in normal and PCa tissues (31 pairs). (C, D) The expression of GOLM1 and KLK3(PSA) in paracancer tissues and PCa tissues of gleason score 3, 4 and 5 grades based on the TMAs (34 PCa patients' samples, 89 points). Scale bar, 50 μm or 500 μm. (E) Correlation analysis of GOLM1 and KLK3 expression in PCa tissues (Pearson *R* = 0.6588). Data were means ± SEM. *p* values were calculated by two‐tailed *t*‐test (B); *p* values were calculated by two‐way ANOVA with Tukey's tests (D). *p < 0.05, **p < 0.01, ****p < 0.001.

Given the significant role of AR signalling in the progression of PCa, a scatter‐correlation analysis was conducted between *GOLM1* and *AR*, as well as its target genes (*KLK3*, *NKX3‐1* and *TMPRSS2*) using data from TCGA‐PRAD. The findings indicated that while *GOLM1* gene expression did not exhibit a direct correlation with AR gene, it was notably correlated with AR's target genes, *KLK3*, *NKX3‐1* and *TMPRSS2* (Pearson *R*: 0.57, 0.62 and 0.36, respectively, as shown in Figure 4A). *KLK3* is the most representative target gene of AR, and mainly encodes prostate‐specific antigen (PSA).^18^ In 31 local PCa cases, the mRNA expression of *GOLM1* and *KLK3* was detected in PCa tissues, and both were remarkably higher than in paracancer tissues, indicating similar expression patterns (Figure 4B). In order to confirm the correlation between GOLM1 and KLK3, we detected GOLM1 and KLK3 on the TMAs, respectively. According to the results, both GOLM1 and KLK3 expression increased with the escalation of PCa Gleason score (Figure 4C,D). Of note, GOLM1 and KLK3 expression in PCa tissues were significantly correlated (Pearson *R* = 0.6588, Figure 4E).

**FIGURE 5:**
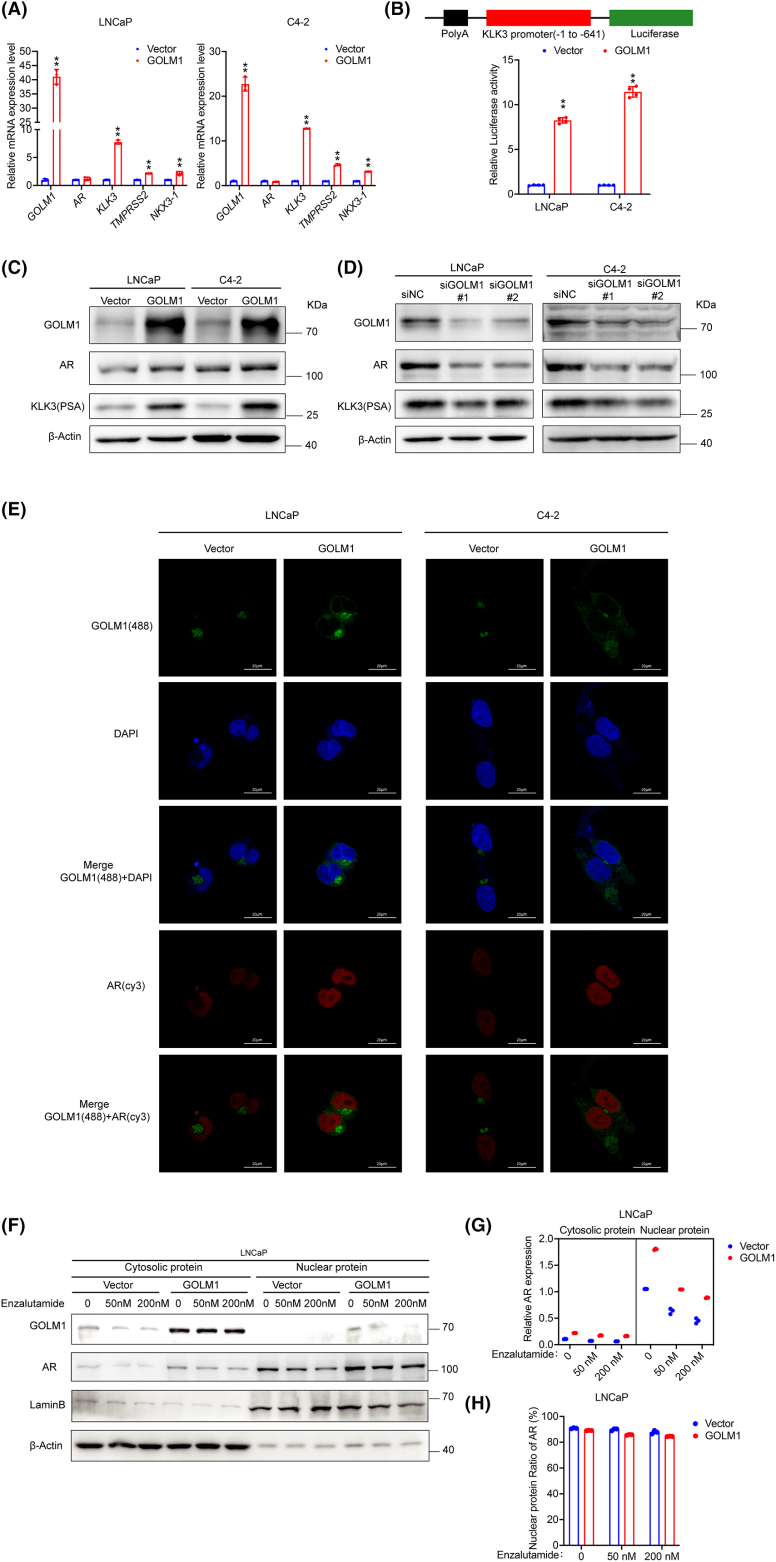
GOLM1 promotes AR protein expression and AR‐driven transcriptional activity. (A) Real‐time RT‐PCR analysis of *AR* and its target genes (*KLK3*, *TMPRSS2*, and *NKX3.1*) in stable GOLM1‐overexpressing and control LNCaP and C4‐2 cells. (B) *KLK3*‐promoter (−1 to −641) luciferase reporter assay in stable GOLM1‐overexpressing and control cells. (C, D) Western blot analysis of the protein expression of AR and its target KLK3(PSA) in stable GOLM1‐overexpressing cells, GOLM1‐silencing cells and control cells. (E) Immunofluorescence analysis of AR expression and nuclear translocation in stable GOLM1‐overexpressing and control cells. Scale bar, 20 μm. (F) Western blot analysis of cytosolic and nuclear AR protein in stable control and GOLM1‐overexpressing LNCaP cells treated with Enzalutamide (0, 50 and 200 nM). (G) Relative expression level of AR in cytosolic and nuclear. (H) Nuclear protein ratio of AR. Data were means ± SEM. *p* values were calculated by Multiple unpaired *t*‐tests (A, B); *p* values were calculated by three‐way ANOVA (G) and two‐way ANOVA with Tukey's multiple comparisons tests (H).*p < 0.05, **p < 0.01.

To further determine the regulatory relationship between GOLM1 and AR signalling. Our study first detected GOLM1 expression in AR activated by dihydrotestosterone^19^ (an AR ligand) or inhibited by enzalutamide^20^ (an AR antagonist) in LNCaP cells. The results suggested that the expression of GOLM1 is not regulated by AR signalling and does not exist as a target gene of AR like KLK3 (Figure S2A–D). Meanwhile, quantitative RT‐PCR was used to evaluate whether GOLM1 regulates AR signalling in LNCaP‐GOLM1 and C4‐2‐GOLM1. The data indicated that the *AR* mRNA expression in GOLM1‐overexpressing PCa cells and control cells was the same, but its target genes (*KLK3*, *TMPRSS2* and *NKX3‐1*) mRNA expressions were obviously upregulated in the GOLM1‐overexpressing PCa cells in comparison to control cells (Figure 5A). A KLK3‐promoter (−1 to −641) luciferase reporter assay also confirmed GOLM1 expression could increase AR target gene transcription (Figure 5B). Next, we continued to detect the levels of protein expression of AR and downstream target KLK3 when GOLM1 was overexpressed or silenced. According to Western blot results, when GOLM expression is upregulated, AR and KLK3 protein levels also increase (Figure 5C). Conversely, when GOLM was silenced, the protein levels of AR and its target gene KLK3 also decreased accordingly (Figure 5D). Immunofluorescence analysis showed AR expression and nuclear translocation in GOLM overexpressing cells LNCaP‐GOLM1 and C4‐2‐GOLM1 were significantly increased compared to the control group cells (Figure 5E). Western blot analysis with cytosolic and nuclear protein showed there was significantly increase of AR nuclear expression in LNCaP‐GOLM1 versus LNCaP‐Vector cells when treated with Enzalutamide (50–200 nM) (Figure 5F,G), yet there was no significant difference in the proportion of AR in the nucleus and cytoplasm compared to the control group (Figure 5H). This indicates that GOLM1 cannot promote the nuclear translocation of AR. These data provide strong evidence to support that GOLM1 is an important positive regulator for AR‐driven transcriptional activation and its nuclear expression.

### 3.5 GOLM1 increases AR protein level and AR‐driven transcriptional activity by interacting with PSMD1

**FIGURE 6:**
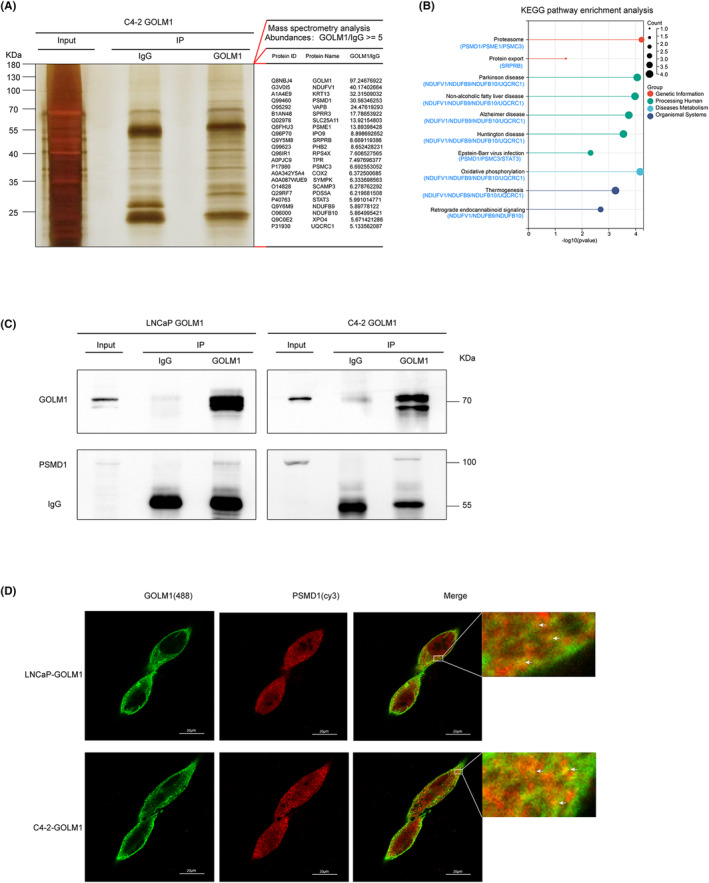
GOLM1 interacts with proteasome 26S subunit, non‐ATPases 1 (PSMD1) in PCa cells. (A) Mass spectrometry analysis of GOLM1‐interacted proteins. Silver stainning of IgG and anti‐GOLM1 Co‐immunoprecipitation samples from stable GOLM1‐overexpressing C4‐2 cells, and the bands were analysed by Mass spectrometry. Twenty‐three candidate molecules interacting with GOLM1 were identified (Abundances: GOLM1/IgG >= 5), detailed in the Table S4. (B) KEGG pathway enrichment analysis of 23 identified GOLM1 interactors. (C, D) Co‐immunoprecipitation analysis and Co‐localization analysis of GOLM1 and PSMD1 in stable GOLM1‐overexpressing LNCaP and C4‐2 cells. Scale bar, 20 μm.

To look into the molecular process through which GOLM1 controls the transcriptional activity of AR, we conducted mass spectrometry analysis to identify interacting molecules of GOLM1 in AR‐positive C4‐2‐GOLM1 cells. A total of 23 candidate molecules were identified through this analysis, with abundances of GOLM1/IgG greater than or equal to 5, as depicted in Figure 6A. Detailed information regarding these molecules can be found in Table S4. The result of KEGG pathway enrichment analysis showed that the 23 candidate molecules were mainly related to proteasome, protein export, oxidative phosphorylation, Parkinson disease, Alzheimer disease and other vital life activities and diseases (Figure 6B). The ubiquitin‐proteasome system (UPS) is crucial for transcription and nuclear receptor transcription in particular.^21^, ^22^ Previous research reported AR and its transcription activity are also regulated by UPS. The UPS is acknowledged as play an essential role in degradation of co‐repressors and transcription complex of AR to promote AR activation and recycling, further enhancing AR‐driven transcriptional activity.^23^ Through the analysis of mass spectrometry data, it was determined that PSMD1, a proteasome 26S subunit non‐ATPase, plays a crucial role in interacting with GOLM1 and regulating proteasome function. Co‐immunoprecipitation assays conducted in GOLM1‐overexpressing LNCaP and C4‐2 cells demonstrated a robust interaction between GOLM1 and PSMD1 (Figure 6C). Additionally, immunofluorescence analysis revealed that GOLM1 and PSMD1 are extensively colocalized in cytoplasm adjacent to the nucleus in GOLM1‐overexpressing LNCaP cells and C4‐2 cells (Figure 6D). Based on the above results, PSMD1 is a key target molecule that interacts with GOLM1 and could be essential to the control of AR‐driven transcriptional activity and PCa development.

**FIGURE 7:**
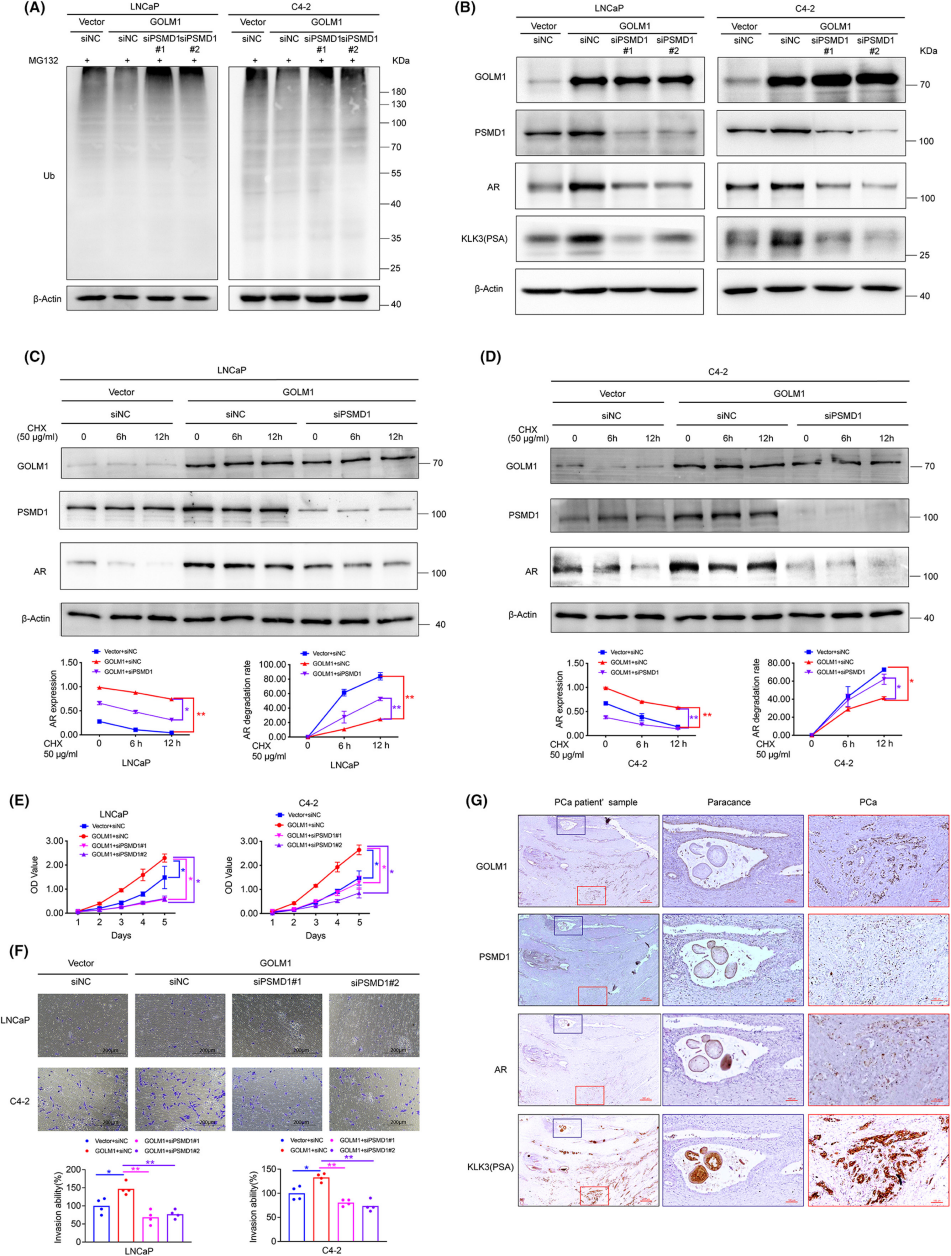
GOLM1 promotes AR‐driven transcriptional activity and PCa progression relies on interaction with PSMD1. (A, B) Western blot analysis of the levels ubiquitylated proteins, AR and its target KLK3(PSA) in stable GOLM1‐overexpressing LNCaP and C4‐2 cells transfected with siNC, siPSMD1#1 and siPSMD1#2. (C, D) Stable control and GOLM1‐overexpressing LNCaP and C4‐2 cells transfected with siNC or siPSMD1 and treated with CHX (50 μg/mL) for 0, 6 and 12 h. AR protein stability was analysed by Western blotting. (E, F) CCK8 assays analysis of cell viability and transwell assays analysis of cell invasion ability were performed in the above‐mentioned cells. (G) Immunohistochemistry analysis of GOLM1, PSMD1, AR and KLK3(PSA) in paracancer tissues and PCa tissues. Scale bar, 100 μm, 200 μm or 500 μm. Data were means ± SEM. Data were means ± SEM. P values were calculated by two‐way ANOVA with Sidak multiple comparisons tests (C–E); *p* values were calculated by one‐way ANOVA with Tukey's multiple comparisons tests (F). *p < 0.05, **p < 0.01.

PSMD1 is a component of the 26S proteasome's 19S regulatory complex, and its main function is to control substrate binding and recognition.^24^ To investigate the involvement of PSMD1 in UPS and GOLM1‐mediated AR signalling, PSMD1 was knocked down using PSMD1‐specific small interfering RNAs (siPSMD1#1 and siPSMD1#2) in GOLM1‐overexpressing AR prositive PCa cell. Western blot results showed that overexpression of GOLM1 significantly decreased ubiquitination accumulation, while silencing PSMD1 restored ubiquitination levels (Figure 7A). These findings suggest that GOLM1 may enhance proteasome activity by activating PSMD1. In order to explore the correlation between PSMD1 and GOLM1‐mediated AR signalling, we conducted Western blot analysis to measure the protein levels of AR and its target KLK3. Our findings indicated that silencing of PSMD1 counteracted the increased expression of AR and KLK3 in cells overexpressing GOLM1 (Figure 7B). By treating with CHX and detecting the lifespan of the AR protein, it was found that GOLM1 could partially inhibit the degradation of the AR protein, and this regulatory process depends on the interaction between GOLM1 and PSMD1 (Figure 7C,D). Additionally, the inhibition of PSMD1 disrupted GOLM1's ability to enhance PCa cell proliferation and invasion (Figure 7E,F). The results indicated that GOLM1‐mediated AR signalling activation and its oncogenic functions were achieved by interaction with PSMD1. This conclusion was substantiated by our examination of PSMD1 and AR signalling components in a sample of six prostate cancer patients exhibiting elevated GOLM1 expression. Immunohistochemical staining results revealed consistent expression patterns and spatial co‐localization of GOLM1, PSMD1, AR and KLK3 (Figure 7G). Taken together, GOLM1 targets PSMD1 to enhance proteasome activity has a prominent function on regulation of AR protein level and AR‐driven transcriptional activity and accelerating PCa progression.

## 4 DISCUSSION

The newly identified tumour‐promoting gene GOLM1 is involved in multiple types of tumours, such as hepatocellular carcinoma, oesophageal carcinoma, lung cancer and others.^10^, ^25^, ^26^ Although it has been reported that GOLM1 is a novel biomarker of PCa, the relevant mechanism and its biological function remain unclear. Here, our findings indicated that GOLM1 was strongly associated with PCa progression and poor prognosis, and its oncogenic function was demonstrated in PCa cells and xenografts as well. In mechanism, GOLM1 have positive effects on the regulation of the level of AR protein and its transcriptional activity. We further identified PSMD1 interacted with GOLM1 and was a key node molecule in GOLM1‐mediated AR signalling. GOLM1 enhanced proteasome activity by interacting with PSMD1, thereby increasing AR signalling and promoting PCa progression. Based on these data, we conclude that GOLM1 uses a new mechanism to play an oncogenic function in PCa.

GOLM1, a Golgi membrane protein, is predominantly expressed in epithelial cells and exhibits increased expression levels in viral infections and malignant diseases.^27^ GOLM1 is a type II transmembrane protein with a long C‐terminal domain and a short transmembrane region at the N end.^28^ It harbours a signal peptidase cleavage site located between amino acids 28 and 29, along with an N‐myristoylation consensus sequence at the N‐terminus.^27^ This structural composition suggests potential involvement in signal transduction and protein phosphorylation processes. Previous studies and pan cancer analysis of TCGA data both have indicated that GOLM1 is upregulated in multiple tumours, suggesting that GOLM1 may be a key regulator for malignant progression. According to the previous research, GOLM1 can move from the trans‐Golgi network into the cytosol, where it can affect the cell surfaces recycling of EGFR/RTK, which is known to be essential for the development of hepatocellular carcinoma.^10^ Additionally, our recent investigation has revealed that GOLM1 is capable of activating the PI3K‐AKT mTOR signalling pathway downstream of RTK in PCa cells.^14^ Given the diverse pathogenesis of PCa, it is hypothesized that GOLM1 may possess novel, unidentified molecular functions and mechanisms in PCa. This study provides additional evidence supporting the positive association between increased GOLM1 expression and the advancement of PCa, as well as unfavourable prognosis, utilizing local TMAs data and TCGA data (Figure 1). The in vitro and in vivo functional studies demonstrate that GOLM1 has a significant promoting effect on PCa development (Figures 2 and 3). Our findings provide further evidence to support that GOLM1 is a clinically and functionally relevant factor in PCa progression.

Excitingly, we for the first time discovered GOLM1 was involved in regulation of AR signalling, and demonstrated that GOLM1 could promote AR protein level and its downstream target genes expression. (Figures 4 and 5). This discovery is very meaningful. AR signalling is crucial in both the onset and advancement of PCa, and its dysregulation is a primary driver of resistance to castration treatment.^4^ One interesting question is how GOLM1 regulates AR protein expression and AR‐driven transcriptional activity. Our results showed that although GOLM1 does not affect the AR mRNA level, it can promote the protein level expression of AR (Figure 5), indicating that GOLM1 may be involved in the regulation of the translation process or protein stability of AR. We further confirmed that although GOLM1 cannot directly affect the AR nuclear translocation, it can significantly promote AR nuclear expression (Figure 5). We all know AR enters the nucleus after combing with ligands and binds to androgen‐response elements (AREs) in the target gene's promoter region, which is a key step in the transcription of downstream genes initiated by AR.^29^, ^30^ More than 150 transcriptional co‐regulators interact with the promoter region to open the chromatin structure and start the transcription process. Some of these coregulators function as co‐repressors or co‐activators, respectively, to increase or decrease the AR‐driven transcriptional activity.^4^ Additionally, certain coregulators possess enzymatic capabilities that enable them to modify other proteins within the complex through processes such as phosphorylation, methylation, acetylation or ubiquitination.^31^ In cancer cells, GOLM1 has the ability to be released into the cytoplasm and acts as a molecular chaperone to interact with various tumour‐related molecules such as EGFR, RTK, MMP2, MMP7 and B3‐H7, exerting a pro cancer effect.^10^, ^32^, ^33^, ^34^ Thus, we speculate that GOLM1 may interact with some transcriptional co‐regulators of AR, thereby promoting AR‐driven transcriptional activation.

Next, we screened 23 molecules interacting with GOLM1 by mass spectrometry analysis, and KEGG pathway enrichment analysis indicated that they were mainly related to proteasome, protein export, oxidative phosphorylation, Parkinson disease, etc. (Figure 6). Among them, proteasomes are most closely associated with AR transcriptional regulation. The ubiquitin‐proteasome system (UPS) is necessary for regulating transcription, particularly in the context of nuclear receptor transcription. The AR, a nuclear receptor, is one such transcription factor whose activity is modulated by UPS‐mediated mechanisms.^23^, ^35^ The AR/NCOR1 complex is known to repress AR‐driven transcriptional activity, but it can be degraded by UPS to restore AR‐driven transcriptional activity by recruiting p300 and other AR coactivators.^36^ Noncanonical polyubiquitination of AR by RNF6 can attract AR coactivators with ubiquitin‐binding domains, such as ARA54.^37^ AR transcription complex (AR‐GRIP1‐CBP) binging KLK3 promoter is transitory and cyclical, and associated with 19S proteasome regulatory sub‐complex.^38^ The degradation of transcriptional complexes by proteasomes not only promotes the AR recycling but also makes ARE available for the binding of fresh AR ligated with androgens, allowing transcription to proceed smoothly and continuously.^23^ In summary, the UPS is essential for the formation of the initiation complex because it facilitates the nuclear localization of AR, eliminates co‐repressors, and maintains AR‐driven transcriptional activity by clearing the initiation complex and starting new transcription cycles.

Ultimately, we identified PSMD1 as a key interacting target of GOLM1, and demonstrated that GOLM1 increases AR protein expression as well as AR‐driven transcriptional activity and promotes the PCa progression mainly by interacting with PSMD1 (Figures 6 and 7). PSMD1, as a constituent of the 19S regulatory complex of the 26S proteasome, is essential for the breakdown of ubiquitinated proteins that are implicated in several biological processes.^39^, ^40^ Within the proteasome, PSMD1 acts as a receptor for ubiquitin, binds peptide substrates, and facilitates gate opening following posttranslational modifications such as SUMOylation, N‐acetylation, succinylation and S‐glutathionylation.^41^ PSMD1 has been identified as an oncogenic factor in various tumours, including breast cancer^24^ and chronic myeloid leukaemia.^42^ The reports indicated PSMD1 promotes NF‐κB protein stability and transcriptional activity in chronic myeloid leukaemia and modulates the degradation of the p53 protein, which contribute to the proliferation of breast cancer cells.^24^, ^42^ Our results also found that GOLM1 enhances the stability of the AR protein through the interaction with PSMD1 (Figure 7). According to our research, PSMD1 plays a pivotal role as a target molecule for GOLM1 in facilitating a pro‐cancer function in PCa. GOLM1 boosts proteasome function through its interaction with PSMD1, leading to its activation. Once the activated proteasome translocates into the nucleus, it facilitates the degradation of co‐repressors of AR, thereby releasing and activating AR. Additionally, the ubiquitination process results in the degradation of the transcriptional regulatory complex within the promoter region of AR target genes, thereby promoting AR recycling and creating opportunities for new transcription factor binding. Therefore, GOLM1 promote AR expression and enhance its transcriptional activity by interacting with PSMD1.

In summary, our study has shown that GOLM1 serves as a novel promoter of PCa progression and is linked to a poor prognosis. GOLM1 interacts with PSMD1 to enhance UPS activity, leading to increased AR protein expression and AR‐driven transcriptional activity, ultimately promoting PCa progression. PCa patients may benefit from GOLM1 as a prognostic marker and therapeutic target. Inhibitors targeting GOLM1 expression, in combination with other inhibitors of AR signalling, may offer a promising strategy for treating PCa.

## AUTHOR CONTRIBUTIONS

**Guang Yan:** Conceptualization (equal); data curation (equal); visualization (equal); writing – original draft (equal). **Tianhang Zhu:** Conceptualization (equal); data curation (equal); visualization (equal); writing – original draft (equal). **Jiawei Zhou:** Conceptualization (equal); data curation (equal); visualization (equal); writing – original draft (equal). **Xia Li:** Data curation (equal); methodology (equal). **Zonghua Wen:** Data curation (equal); methodology (equal). **Bahaerguli Miuhuitijiang:** Methodology (equal); validation (equal). **Zhiyong Zhang:** Methodology (equal); validation (equal). **Yuejun Du:** Formal analysis (equal); methodology (equal). **Chengyao Li:** Writing – review and editing (equal). **Xiaojun Shi:** Project administration (equal); supervision (equal); writing – review and editing (equal). **Wanlong Tan:** Project administration (equal); supervision (equal); writing – review and editing (equal).

## FUNDING INFORMATION

This work was supported by grants from the National Natural Science Foundation of China (No. 82073162 and No. 82372867), China Postdoctoral Science Foundation (2022M711520). Supported by Pudong Institute of Clinical Chinese Medicine (YC‐2023‐0609), The ‘Fourteenth Five‐Year Plan’ Traditional Chinese Medicine Specialty Project for the Construction of Andrology Departments in TCM (ZYTSZK1‐4).

## CONFLICT OF INTEREST STATEMENT

We declare no conflicts of interest.

## Supporting information


Figures S1–S2.

Table S1.

Table S2.

Table S3.

Table S4.

## Data Availability

The data that support the findings of our study are available from the corresponding author upon reasonable request.
