# Supplementary material for: GOLM1 promotes prostate cancer progression via interaction with PSMD1 and enhancing AR‐driven transcriptional activation
Source: J Cell Mol Med. 2024 Oct 29;28(20):e70186. doi: 10.1111/jcmm.70186 (PMC11520440; doi:10.1111/jcmm.70186)
Supplement: Supplementary file 1 — Figures S1–S2. [file JCMM-28-e70186-s005.zip › Supplemental Legends.docx]

**Supplemental Figure Legends**

Supplemental Figure 1. *GOLM1* mRNA expression in GOLM1- overexpressing or silencing cells. (A, B) Real-time RT-PCR analysis of *GOLM1* mRNA level in GOLM1-overexpressing stable cells, GOLM1-silencing cells and control cells. Data were means ± SEM. P values were calculated by Multiple unpaired t tests (A). P values were calculated by by one-way ANOVA with Dunnett’s multiple comparisons tests (B). ***p* < 0.01.

Supplemental Figure 2. GOLM1 is not regulated by AR signaling. (A, B) Real-time RT-PCR analysis of GOLM1 and KLK3 in LNCaP cells treated with Dihydrotestosterone (0, 50 nM,100 nM, 500 nM,1 μM) or treated with Enzalutamide (0, 10 nM, 50 nM,100 nM, 200 nM). (C, D) Western blot analysis of the protein levels of GOLM1, AR and KLK3(PSA) in LNCaP cells treated with Dihydrotestosterone (0, 50 nM,200 nM) or treated with Enzalutamide (0, 50 nM, 200 nM). Data were means ± SEM. P values were calculated by by one-way ANOVA with Tukey’s multiple comparisons tests. ***p* < 0.01, *****p* < 0.001.
