# Supplementary figures and images for: GOLM1 promotes prostate cancer progression via interaction with PSMD1 and enhancing AR‐driven transcriptional activation
Source: J Cell Mol Med. 2024 Oct 29;28(20):e70186. doi: 10.1111/jcmm.70186 (PMC11520440; doi:10.1111/jcmm.70186)

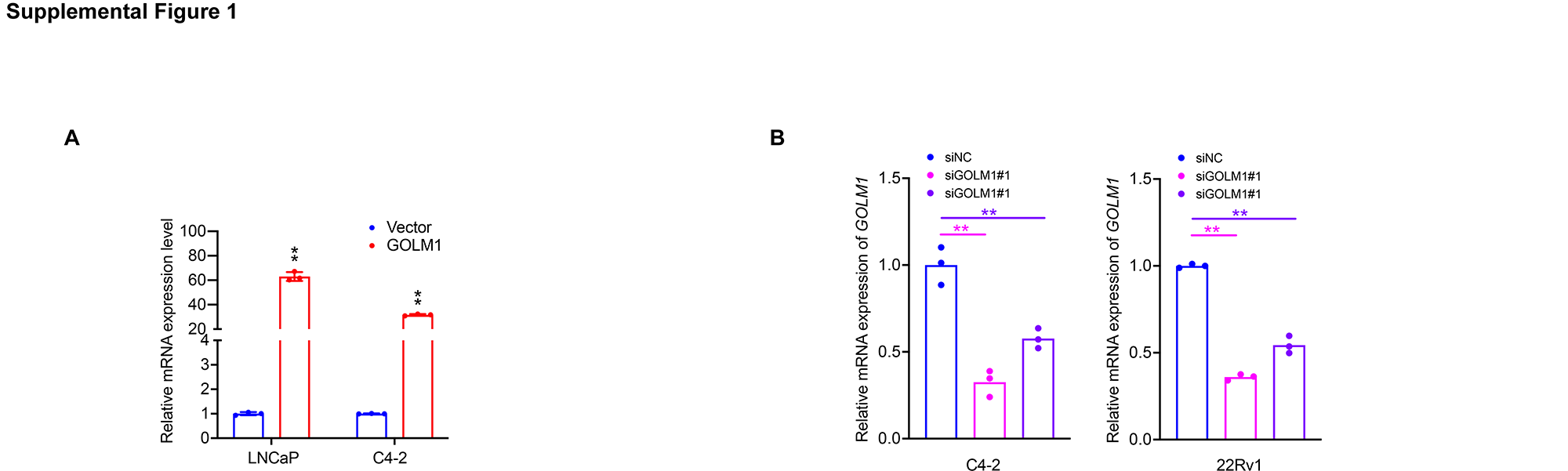

Supplement: Supplementary file 1 — Figures S1–S2. [file JCMM-28-e70186-s005.zip › Supplemental Figure 1.tif]

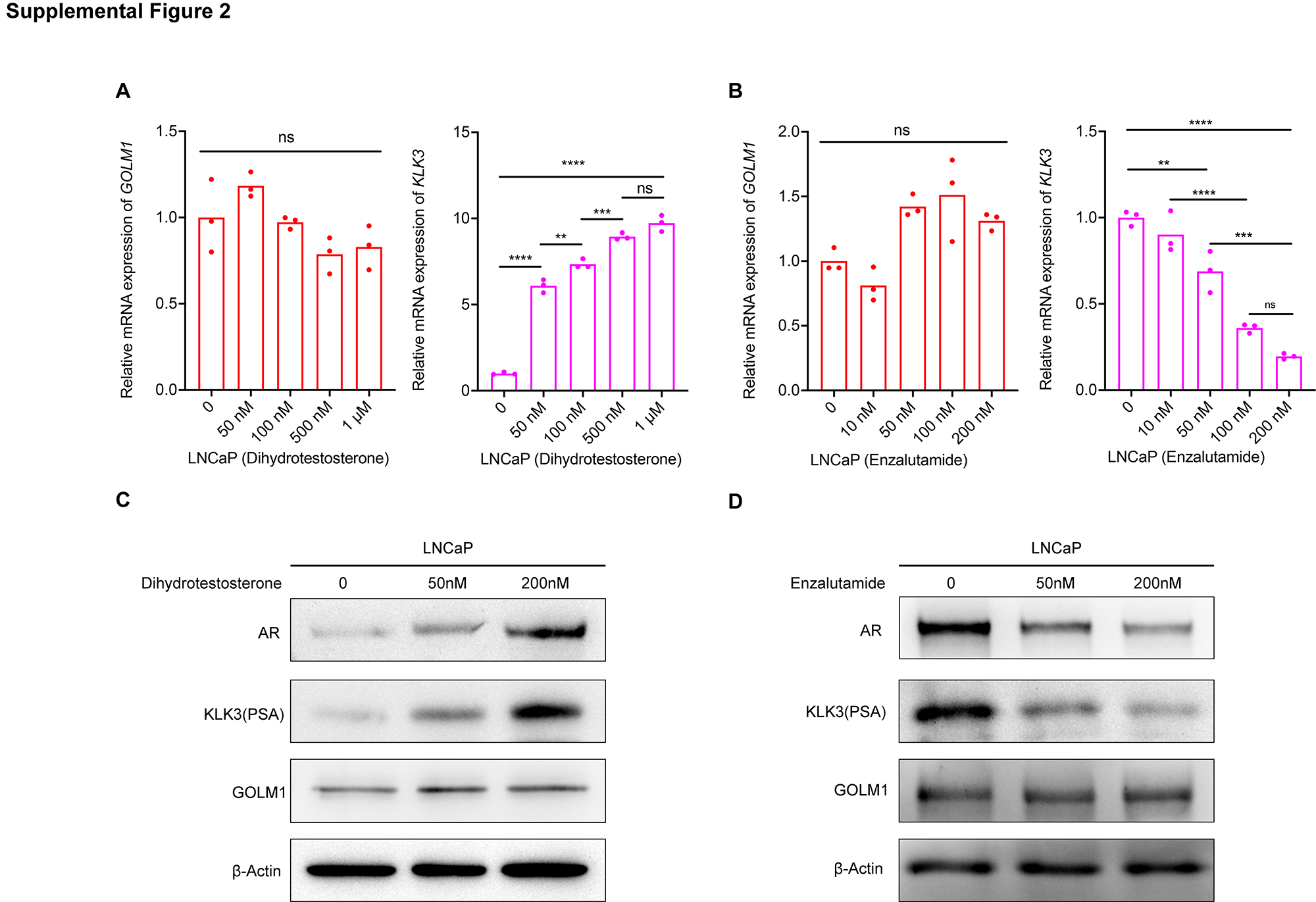

Supplement: Supplementary file 1 — Figures S1–S2. [file JCMM-28-e70186-s005.zip › Supplemental Figure 2.tif]
